# Nurses’ practices in stroke-related dysphagia in low- and middle-income countries

**DOI:** 10.4102/curationis.v47i1.2499

**Published:** 2024-03-28

**Authors:** Kirsten Rowe, Maria N. du Toit, Sarveshvari B. Pillay, Esedra Krüger

**Affiliations:** 1 Department of Speech-Language Pathology and Audiology, Faculty of Humanities, University of Pretoria, Pretoria, South Africa

**Keywords:** nursing practices, stroke, dysphagia, scoping review, low- and middle-income countries

## Abstract

**Background:**

Nurses are often required to perform dysphagia screening prior to oral intake by people following stroke. Previous studies report limited knowledge of nurses in identifying symptoms of post-stroke dysphagia.

**Objective:**

To explore existing literature regarding nurses’ practices and knowledge in the identification and management of post-stroke oropharyngeal dysphagia (OPD) in low- and middle income countries (LMICs).

**Method:**

A scoping review was conducted according to the PRISMA-ScR guidelines. Studies were retrieved from PubMed, Scopus, EBSCOhost (CINAHL and Health source: Nursing and Academic edition), Web of Science Core collection, and Cochrane libraries. No time frame was applied, and all included studies were screened according to predefined eligibility criteria.

**Results:**

Eight studies were included from 1 792 initial hits. Studies described nursing practices in acute care pertaining to identification and management of stroke-related dysphagia in LMICs. Increased knowledge was reported in nurses who had greater clinical experience in managing patients with dysphagia. Needs for training relating to dysphagia management and opportunities for interprofessional collaboration with speech-language therapists (SLTs) were identified. Contextual barriers specific to LMICs impacting on optimal nursing management of dysphagia included heavy workloads, staff-shortages and time constraints.

**Conclusion and contribution:**

Eight studies described nurses’ practices and identified needs for the improvement of nurses’ dysphagia care in LMICs. This scoping review highlighted the urgency for further research in dysphagia management that provides creative, contextually relevant solutions for improved protocols and training of health care professionals. Findings may be valuable for the multidisciplinary team involved in post-stroke dysphagia care.

## Introduction

Oropharyngeal dysphagia (OPD) is a common complication after stroke, with a highly variable frequency of occurrence (Daniels, Huckabee & Gozdzikowska 2019; El-Koussy et al. 2016). Post-stroke, OPD has been associated with increased mortality and morbidity because of malnutrition, dehydration and aspiration pneumonia (Suntrup-Krueger et al. 2017). Appropriate and timeous management of stroke-related OPD across health care professions may reduce the occurrence of aspiration pneumonia (Bray et al. 2016). Nurses are often the first health care professionals who have contact with patients admitted with a stroke (Khoja 2018). This positions nurses to identify signs of OPD and initiate referral to the appropriate professionals, including speech-language therapists (SLTs), before further complications arise (Liu et al. 2016). The occurrence of stroke and associated comorbidities continue to be highly prevalent in low- and middle-income countries (LMICs), such as South Africa (Andrews & Pillay 2017). In LMICs, there is a shortage of skilled allied health care professionals such as SLTs, who can perform timely, comprehensive bedside swallowing examinations (Pierpoint & Pillay 2020). The Health Professions Council of South Africa (HPCSA) has only 2643 SLTs registered (Pillay et al. 2020). A limited workforce may negatively affect patient management and can result in general health complications (Robbertse & De Beer 2020).

Previous studies reported limited knowledge of nurses in identifying symptoms of post-stroke OPD, because of a lack of information presented in tertiary education settings (Khoja 2018; Seedat & Strime 2021). Because of insufficient undergraduate and in-service training, nurses have better knowledge of the symptoms rather than the complications and management of OPD, negatively affecting overall practices (Knight et al. 2020). A swallow disorder may result in food or liquid entering the lungs and causing pneumonia. Most stroke-related pneumonia is because of OPD and subsequent aspiration of ingested food or liquid (Feng et al. 2019). Screening and training programmes to prepare nurses working in an acute stroke setting are frequently described in high-income countries (Pierpoint & Pillay 2020); however, the same cannot be said of LMICs (Robbertse & de Beer 2020) warranting further research. This is particularly crucial given that nurses often bear the responsibility of providing post-stroke care, owing to shortages of SLTs.

Role sharing between nurses and other allied health professionals such as SLTs, is recommended to address the shortage of SLTs and the need for timeous OPD identification to ensure patient-centred care in acute settings (Pierpoint & Pillay 2020; Seedat & Strime 2021). Role sharing between nurses and SLTs refers to the comprehension, appreciation and sharing of one another’s scope of practice and professional responsibilities (Pierpoint & Pillay 2020). There is guidance in high-income countries, such as the United Kingdom, regarding structured information sharing relating to the management of patients post-stroke (Barnard et al. 2021); however, dysphagia guidelines and services need to be better streamlined in LMICs. There is a need to explore the knowledge and practices of nurses in stroke-related OPD management within LMICs. The aim of this study was thus to integrate available research and identify caveats in the current literature on nursing knowledge and practices in stroke-related OPD in LMICs.

## Method

### Protocol development

This scoping review was conducted and documented according to the PRISMA-ScR (Preferred Reporting Items for Systematic review and Meta-Analysis extension for Scoping Reviews) guideline, a widely used framework that encourages transparent and comprehensive reporting and comparing of scoping reviews (Tricco et al. 2018).

### Search strategy

Studies were retrieved from the following five electronic databases: PubMed, Scopus, EBSCOhost (CINAHL and Health source: Nursing and Academic edition), Web of Science Core collection, and Cochrane libraries, in April and May 2021. No time frame was applied to the search in order to include all relevant studies. All studies listed during the search were screened according to predefined criteria for inclusion. The following Boolean phrase and/or keyword combinations were used (Appendix 1):

- ‘Knowledge’ AND ‘nurses’ AND ‘stroke’ AND ‘dysphagia’
- ‘Practices’ AND ‘nurses’ AND ‘stroke’ AND ‘dysphagia’
- ‘Collaboration’ AND ‘nurses’ AND ‘stroke’ AND ‘dysphagia’
- ‘Knowledge’ AND ‘nurses’ AND ‘stroke’ AND ‘dysphagia’ AND ‘low- and middle-income countries’

### Eligibility criteria

#### Inclusion criteria

Studies conducted on current practices of nurses in stroke-related OPD in LMICs were included. Grey literature was also included to ensure a comprehensive search and appraisal of all available information sources. Only studies involving adult participants (>18 years) with post-stroke OPD as well as studies involving interprofessional collaboration or teamwork between nurses and SLTs, in stroke-related OPD, were included.

#### Exclusion criteria

Studies conducted in high-income countries or published in a language other than English were excluded in order to minimise resource challenges in terms of time and expertise in non-English languages. If participants were not qualified nurses or if nursing practices described were not specifically related to the assessment or treatment of OPD, or post-stroke care, studies were excluded. Review articles and systematic reviews were also excluded.

### Study selection

DistillerSR software was used to select and screen studies obtained from the keyword search (Evidence Partners 2021). The initial database search yielded 1792 results. Duplicate articles were excluded (*n* = 178) whereafter title screening was performed with remaining articles (*n* = 1614). Abstract screening was performed with the remaining 281 articles, according to inclusion and exclusion criteria. During the abstract screening, 222 articles were found to be unrelated and were excluded. Twenty five percent of the 281 abstracts screened by the first author (K.R.) were randomly selected and screened by the third author (M.dT.). A 90% similarity was achieved, and the discrepancy was addressed prior to full-text screening. The 59 articles that qualified for full-text review were individually obtained and screened by the first author (K.R.) (Table 1).

**TABLE 1:** Screening of full-texts with reasons for exclusion (*N* = 51).

Reason for exclusion	*n*	Total (%)
Study conducted in a high-income country	24	47.0
Participants do not work with patients with stroke-related OPD	22	43.2
Participants are not qualified nurses	17	33.3
Patients with stroke are younger than 18 years of age	11	21.5
Nursing practices not related to the management of OPD	8	15.6
Commentary without original results	3	5.8
Insufficient information to assess methodological quality	3	5.8
Full-text report could not be retrieved	2	3.9
Review article or systematic review	1	1.9
Full-text not available in English	1	1.9

OPD, Oropharyngeal dysphagia.

**FIGURE 1:**
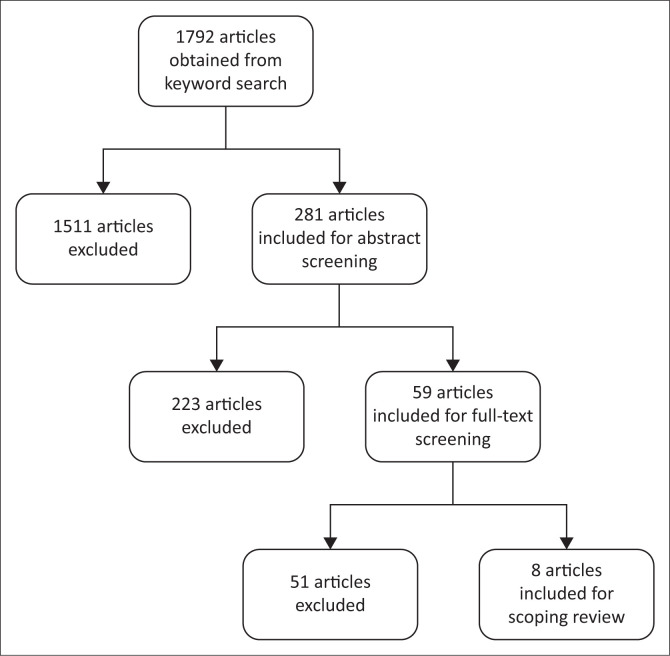
Outcome of the search process and number of studies included in scoping review.

Twenty five per cent of the 59 full-text articles were screened by the second author (B.P.) prior to data extraction. Inconsistencies were discussed and resolved through reflection on inclusion criteria. Eight articles met the predefined criteria and were included for data extraction. The study selection process (Figure 1) was performed according to the PRISMA-ScR guidelines.

### Data collection and analysis

The first author (K.R.) extracted the following data from the eight studies: title, authors, date of publication, study design, sampling strategy, setting, geographic location of the primary and additional site, total number of participants (including controls), American Speech-Language-Hearing Association (ASHA) level of evidence (ASHA 2004) and specific details regarding nursing practices and knowledge reported (Table 2).

**TABLE 2:** Study characteristics and level of evidence (*N* = 8).

Article	Study features				Number of participants (including controls)	Location				Level of evidence (ASHA 2004)	Study variables and outcomes		
	Author(s)	Date	Study design	Sampling strategy		Country (LMIC)	Number of sites	Setting	Specific division within main setting		Nursing practices were reported by:	Nursing practices described	Needs identified from practices described
Perceived barriers to compliance with speech-language therapist dysphagia recommendations of South African nurses	Robbertse, A., & De Beer, A.	2020	Cross-sectional survey	Convenience	Case: 81	South Africa Upper middle income	2	Hospital	GNW	III	Nurses themselves	Treatment Monitoring Referral MDT DPC	TKSA HR TM IPC
Nurses’ knowledge of stroke-related oropharyngeal dysphagia in the Eastern Cape, South Africa	Knight, K., Pillay, B., Van der Linde, J., & Krüger, E.	2020	Descriptive survey	Purposive	Case: 130	South Africa Upper middle income	5	Hospital Clinic	AC	III	Nurses themselves	Evaluation Treatment Monitoring DPC	TKSA IPC
Post-stroke dysphagia: An exploration of initial identification and management performed by nurses and doctors	Pierpoint, M., & Pillay, M.	2020	Cross-sectional survey	Non-probability purposive	Case: 25 (21 nurses & 4 doctors)	South Africa Upper middle income	1	Hospital	ICU AC GNW	III	Nurses themselves	AS Evaluation Treatment Referral MDT	TKSA MR IPC
Training in swallowing prevents aspiration pneumonia in stroke patients with dysphagia	Huang, J.Y., Zhang, D.Y., Yao, Yu, Xia, Q. X., Fan, Q.Q.	2006	Prospective cohort study	Purposive	Case: 96	China Upper middle income	1	Hospital	AC	IIb	Another party	Evaluation Treatment DPC	TKSA
Knowledge of nurses regarding dysphagia in patients post-stroke in Namibia	Rhoda, A., Pickel-Voight, A.	2015	Non-experimental survey	Non-probability convenience sampling	Case: 182	Namibia Upper middle income	1	Hospital	GNW	III	Nurses themselves	AS Evaluation Treatment DPC	TKSA
Nursing management of post-stroke dysphagia in a tertiary hospital: A best practice implementation project	Liu, H., Shi, Y., Shi, Y., Hu, R., Jiang, H.	2016	Prospective cohort study	Non-probability	Case: 80	China Upper middle income	1	Hospital	GNW	IV	Another party	AS Treatment Referral MDT DPC	TKSA IPC
Knowledge of dysphagia in stroke among nurses working in tertiary care hospital	Nepal, G.M., Sherpa, M.D.	2019	Descriptive cross-sectional survey	Census	Case: 46	Nepal Lower middle income	1	Hospital	ICU ED GNW	III	Nurses themselves	AS Treatment DPC	TKSA
Clinical validation of the related factors and defining characteristics of impaired swallowing for patients with stroke	Jeng, C., Sheu, P. Y., Chen, C. M., Chen, S. R., Tseng, I. G.	2001	Case-controlled	Convenience	Nurses: 52 Patients: Case: 51 Control: 56	Taiwan Upper middle income	4	Hospital	GNW	IIa	Another party	AS Evaluation	TKSA

Note: Please see the full reference list of the article, Rowe, K., Du Toit, M.N., Pillay, S.B, & Krüger, E., 2024, ‘Nurses’ practices in stroke-related dysphagia in low- and middle-income countries’, *Curationis*, 47(1), a2499. https://doi.org/10.4102/curationis.v47i1.2499, for more information

ASHA, American Speech-Language-Hearing Association; GNW, general neurological ward; MDT, multi-disciplinary team; DPC, daily patient care; TKSA, Training for improved knowledge & skill acquisition; HR, human resources; TM, time management; IPC, interprofessional collaboration; AC, acute care; ICU, intensive care unit; AS, admissions/screening; MR, material resources; ED, emergency department.

To allow for comprehensive evaluation of data, qualitative comparisons were performed regarding how nursing practices and knowledge were reported (i.e., self-reported by survey completion or reported on by another party such as patients or the multi-disciplinary team [MDT]), what were the described nursing practices related to, and the needs identified from nursing practices. An exploratory approach was used to identify keywords and trends in selected studies, before analysis took place (Guest, MacQueen & Namey 2011). Thematic analysis was used to organise and synthesise information extracted from the selected studies as this approach facilitates identification and description of implicit and explicit ideas contained in the data, depicted as various themes (Guest et al. 2011). Codes from keywords and phrases that occurred frequently across selected studies were analysed, compared and contrasted, to generate themes.

## Results

### Study characteristics

All studies used an observational design (*n* = 8) with one case-control study, two cohort studies, and five cross-sectional studies (Table 2). The five cross-sectional studies used surveys to collect data, one study used an implementation project and two used formal testing prior to recording of results. The implementation project involved application of strategies to improve nursing management in OPD and used six audit criteria at baseline and follow-up in order to assess changes in nursing practice (Liu et al. 2016). Formal testing included the Burke’s Dysphagia Screening Test to identify pneumonia (Jeng et al. 2001) and non-enhanced computed tomography (CT), magnetic resonance imaging (MRI) of the brain, and a swallowing provocation test to confirm dysphagia (Huang et al. 2006). All studies used non-probability purposive or convenience sampling. All studies were conducted in hospitals in upper-middle (*n* = 7) or lower-middle income (*n* = 1) countries. Lower-middle income countries are classified by the World Bank as countries with a gross national income of $1136 and $4465, while upper-middle income countries are classified as countries with a gross national income between $4466 and $13 845 (World Bank Group 2022). Study sample size ranged from 25 to 182 participants, which included qualified nurses working with adult patients who had stroke-related OPD. The studies were rated according to the ASHA level of evidence scale (Table 2) (ASHA 2004). One study (12.5%) achieved a high level of evidence rating (IIa), while another (12.5%) achieved a rating of IIb. Five studies (62.5%) achieved a lower rating of III, and the implementation report, classified as grey literature, achieved the lowest rating of IV.

In five studies (62.5%), perceptions of nurses’ knowledge and nursing practices were self-reported. It should be considered that self-report measures have the potential to introduce response bias and can be susceptible to the influence of environmental conditions. But research further indicates that objective measures may lack individual-level variation and may, therefore, fail to capture the subjective, yet valuable, experiences (Rosenman, Tennekoon & Hill 2011). In three studies (37.5%), nursing practices were reported on by another party (i.e., an MDT member). Nursing practices described across the eight studies included screening for OPD upon admission (*n* = 5; 62.5%), evaluation of swallowing (*n* = 5; 62.5%), treatment including dietary modification, compensatory strategies or swallowing manoeuvres taught to patients (*n* = 7; 87.5%), monitoring safety and efficiency of oral intake (*n* = 2; 25%), referral to relevant MDT members (*n* = 3; 37.5%), and daily patient care related to OPD (*n* = 5; 62.5%). Based on nursing practices described, all eight studies (100%) identified various needs, including further training for improved knowledge and skill acquisition related to identification and management of OPD post-stroke. Other needs identified related to people management such as increased staff numbers to share the workload (*n* = 1; 12.5%), greater material resources to perform appropriate dysphagia screening (*n* = 1; 12.5%), improved time management (*n* = 1; 12.5%) and further interprofessional collaboration opportunities between nurses and SLTs (*n* = 4; 50%).

The following themes were identified across all included studies relating to nurses’ knowledge, attitudes and practices in post-stroke OPD: (1) identification of signs and symptoms and the awareness of complications related to OPD, (2) management of patients with stroke-related OPD, (3) nurses’ experience in stroke care and managing stroke-related OPD, (4) further training needs of nurses in post-stroke OPD management, (5) need for interprofessional collaboration between nurses and SLTs and (6) current barriers to optimal nursing management in stroke-related OPD in LMICs.

#### Identification of Oropharyngeal dysphagia and awareness of related complications

Identification of OPD upon admission was described in six studies and related to whether nurses followed structured screening processes. Nurse-initiated dysphagia screening using any validated tool allowed early initiation of intervention in two studies and assisted in preventing further complications related to dysphagia (Jeng et al. 2001; Liu et al. 2016). Informal swallowing assessments such as observation were performed in four studies, to identify symptoms of OPD following acute stroke (Knight et al. 2020; Nepal & Sherpa 2019; Pierpoint & Pillay 2020; Rhoda & Pickel-Voight 2015). Results from two studies indicated that nurses have moderate knowledge of signs and symptoms in identification of stroke-related OPD (Nepal & Sherpa 2019; Rhoda & Pickel-Voight 2015). Without a formal screening tool and moderate knowledge, clinical signs of OPD, such as inadequate voluntary cough strength and poor vocal quality post-swallow, were neglected (Pierpoint & Pillay 2020). In three studies relating to OPD identification, nurses recognised coughing as an indication of dysphagia and possible aspiration; however, they were unaware of and did not record the risk for silent aspiration (Knight et al. 2020; Nepal & Sherpa 2019; Rhoda & Pickel-Voight 2015). The lack of knowledge related to silent aspiration was identified as an important factor in patient mortality (Nepal & Sherpa 2019). Knowledge of identification of OPD, specifically nurses’ knowledge on OPD complications, was scored low in one study (Knight et al. 2020) and moderate in two studies (Nepal & Sherpa 2019; Rhoda & Pickel-Voight 2015).

#### Management of stroke-related Oropharyngeal dysphagia

Treatment of patients with stroke-related OPD was described in six studies. Knowledge regarding management of stroke-related OPD varied and was described as moderate in one study (Nepal & Sherpa 2019) and poor in another study (Rhoda & Pickel-Voight 2015). Management of patients with stroke-related OPD included the recommendation of enteral or parenteral feeds in one study (Pierpoint & Pillay 2020), use of compensatory swallowing strategies or optimal positioning during meals in two studies (Huang et al. 2006; Pierpoint & Pillay 2020), rehabilitation and swallowing exercises in two studies (Huang et al. 2006; Pierpoint & Pillay 2020) and appropriate referral to SLTs in two studies (Liu et al. 2016; Pierpoint & Pillay 2020). The need to improve nurses’ evidence-based practice in clinical management of stroke-related OPD was identified in two studies (Liu et al. 2016; Nepal & Sherpa 2019).

#### Nurses’ experience in stroke care and managing stroke-related Oropharyngeal dysphagia

Nurses’ knowledge related to identification and management of OPD caused by stroke was associated with nurses’ experience in caring for stroke patients and was not determined by knowledge acquired at a tertiary level (Knight et al. 2020; Nepal & Sherpa 2019; Rhoda & Pickel-Voight 2015). There is a lack of introduction to stroke-related OPD in the nursing curriculum at an undergraduate level (Nepal & Sherpa 2019). Increased exposure to patients with stroke resulted in provision of appropriate care and improvement of nurses’ knowledge and confidence (Knight et al. 2020; Nepal & Sherpa 2019; Rhoda & Pickel-Voight 2015), rather than nurses’ position, qualification or years of experience in general patient care (Rhoda & Pickel-Voight 2015).

#### Training needs in post-stroke Oropharyngeal dysphagia management

A need for further training for improved knowledge and skill-acquisition was described in seven studies (Huang et al. 2006; Knight et al. 2020; Liu et al. 2016; Nepal & Sherpa 2019; Pierpoint & Pillay 2020; Rhoda & Pickel-Voight 2015; Robbertse & de Beer 2020). In three studies, nurses reported they were dissatisfied with their current knowledge of OPD and recognised further training as a need for improved clinical management (Knight et al. 2020; Nepal & Sherpa 2019; Rhoda & Pickel-Voight 2015). In-service training scheduled around nurses’ availability will ensure that training does not create additional work for nurses (Robbertse & De Beer 2020). Nurses reported that both material training tools and practical application of newly acquired information were necessary for carry-over of skills (Robbertse & De Beer 2020). Training in the anatomy and physiology of the swallowing mechanism, and how to feed patients with stroke-related OPD, was directly related to the prevention of aspiration pneumonia in one study (Huang et al. 2006), which should be the minimum knowledge that nurses have to possess to care for patients with stroke-related OPD.

#### Interprofessional collaboration between nurses and speech-language therapists

The need for support regarding appropriate referral to SLTs and increased interprofessional collaboration was identified in five studies (Knight et al. 2020; Liu et al. 2016; Pierpoint & Pillay 2020; Rhoda & Pickel-Voight 2015; Robbertse & De Beer 2020). Interprofessional collaboration optimised referral systems while reducing time constraints faced by nurses working with stroke-related OPD (Knight et al. 2020; Liu et al. 2016; Pierpoint & Pillay 2020; Robbertse & de Beer 2020). Open communication between nurses and SLTs improved patient-centred care as nurses were able to guide SLTs in understanding cultural, spiritual and personal preferences of patients (Knight et al. 2020; Robbertse & de Beer 2020). A lack of collaboration was because of a limited number of SLTs within the treatment setting and subsequent lack of exposure and transfer of skills among nurses and SLTs (Robbertse & de Beer 2020).

#### Barriers to optimal nursing management in stroke-related Oropharyngeal dysphagia in low- and middle-income countries

Optimal identification and management of stroke-related OPD was also impacted by patient-related barriers and workplace concerns in four studies (Knight et al. 2020; Pierpoint & Pillay 2020; Rhoda & Pickel-Voight 2015; Robbertse & de Beer 2020). Heavy workloads, staff shortages and subsequent time constraints were challenges that negatively impacted nurses’ management of stroke-related OPD. Insufficient access to in-service training opportunities, scheduled in accordance with nurses’ availability, also presented a barrier to knowledge and skill acquisition (Knight et al. 2020; Robbertse & de Beer 2020).

## Discussion

This scoping review identifies several nursing practices related to stroke-related OPD. This includes screening for OPD upon admission, evaluating swallowing ability, modifying diets, teaching compensatory strategies or swallowing manoeuvres, monitoring safety of oral intake and referring to relevant members of the multidisciplinary team. However, the effectiveness of these practices may depend on nurses’ knowledge and undergraduate training regarding OPD, as well as their experiences in managing stroke-related OPD. In particular, nurses in LMICs may face additional challenges because of limited resources and few training opportunities.

It is crucial that nurses are knowledgeable and skilled in screening for OPD, to ensure safety, as they are often the primary care providers for stroke patients (Gurevich, Osmelak & Osentoskia 2021). Studies suggest that nurses recognise gaps in their own knowledge and see further training as the best way to increase competence and confidence in managing OPD (Seedat & Strime 2021). An important clinical implication of the study is to use well-designed training programmes, courses and continuous professional development events that are developed and tailored to improve specific dysphagia knowledge and skills in LMICs. In-service training should be dynamic and inclusive of daily patient care to facilitate functional learning and scheduled to avoid adding to workload stress (Robbertse & de Beer 2020; Seedat & Strime 2021).

Although nurses may not always receive training on dysphagia management during undergraduate training, transfer of skills and knowledge from other members of the health care team, particularly SLTs, is essential (Gilbert, Yan & Hoffman 2010). Limited knowledge about OPD and its complications, such as aspiration pneumonia, has been identified among nurses in LMICs and high-income countries alike (Diendéré et al. 2016). Silent aspiration is a particularly concerning complication, as it may go unnoticed by nurses during informal swallowing screenings (Dondorf, Fabus & Ghassemi 2015; Liu et al. 2016). Therefore, integrating a screening sheet into daily nursing progress reports may ensure that all acute stroke patients are formally screened for OPD, which may identify signs of possible aspiration, leading to improved patient outcomes (Liu et al. 2016).

Nurses experienced in OPD management are able to identify life-threatening symptoms and take heed to provide high-quality care to patients following stroke (Abu-Snieneh & Saleh 2018). The use of screening tools allows nurses to confidently identify factors (i.e., a poor oral phase, signs of aspiration and a weak cough) and characteristics such as neurological impairment, mechanical obstruction and limited awareness, which are reliable and valid in diagnosing OPD (Jeng et al. 2001).

Screening tools, such as the South African Dysphagia Screening Tool (SADS), may be useful in identifying patients at risk of OPD following stroke. However, research is required on the feasibility of available tools in LMICs (Ostrofsky & Seedat 2016). Future education for nurses should include formal screening tools and guidelines or protocols for managing stroke-related OPD, as well as education on other aspects of stroke care, such as acute treatment and prevention of complications (Abu-Snieneh & Saleh 2018). Empowering nurses in accurate identification and effective management of stroke-related OPD will not only ensure evidence-based practice in patient-centred care but also support hospital-based SLTs in LMICs who have large caseloads. The implementation of validated dysphagia screening programmes has been identified as a need, specifically in LMICs (Pierpoint & Pillay 2020).

In reality, many hospitals in LMICs have overworked nurses, with a limited number of specialty nurses, attending to large patient loads in settings with limited material resources (Robbertse & De Beer 2020), and thus strict adherence to management protocols may not always be possible. Regular in-service training and support of nurses by other MDT members and hospital management may ensure adherence to the South African Stroke Society (SASS) (Bryer et al. 2010) guidelines in LMICs. Stroke guidelines for optimal patient care in both high-income (Hebert et al. 2016) and LMICs convey similar information, but contextual constraints place additional pressure on nurses and hinder compliance with SLT recommendations. As further identified in the study, SLTs and nurses are called to action to work collaboratively with confidence in each other’s skills to effectively manage patients with stroke-related OPD (Dondorf et al. 2015). Training of interprofessional collaborative practice on an undergraduate level for health care professions is therefore essential to optimise referral systems, reduce time constraints and ensure best practice (Dondorf et al. 2015; Gurevich et al. 2021). Speech-language therapists rely on nursing staff to screen patients presenting with OPD symptoms before referral for a comprehensive swallowing assessment is made (Seedat & Strime 2021). Clarification of professional roles and clear communication channels between nurses and SLTs may assist in achieving agreement about the care of patients with stroke-related OPD (Dondorf et al. 2015). Interprofessional collaboration is an asset in patient-centred care, and it is the responsibility of all members of the MDT to pursue (Hickey & Livesay 2015). Upskilling nurses with continuous education opportunities will not only likely result in improved knowledge and confidence but may also reduce SLTs’ workload and improve patient outcomes (ASHA 2021; Sandhaus et al. 2009). Nurses with training in dysphagia care are more likely to make appropriate referrals to SLTs, reducing the chances of associated complications (Gilbert et al. 2010).

## Study limitations

This scoping review has several limitations that should be acknowledged. Firstly, the sample size was small and the number of sites within each study for data collection were relatively few, which limits the generalisability of the findings. Secondly, many studies used a non-randomised/non-probability sampling method, which limits external validity and generalisability. Thirdly, five of the studies used a survey design, which is subject to bias because of self-reported data and may not always provide an accurate depiction of nurses’ knowledge and performance. Additionally, 1 of the 59 studies were excluded because of being in a language other than English, which may have introduced a language bias.

## Future research

Various needs have been identified in the care of patients with stroke-related OPD, and further research is required to address these needs. One area that requires attention is the need for education and continued professional development, as well as collaboration among health care professionals working with stroke-related OPD. This is a common need identified in both low- and high-income countries requiring ongoing research effort (Cameron, Gignac & Ahmad 2014).

Another area that requires investigation is the implementation of in-service education and support regarding interprofessional collaboration between nurses and SLTs in LMICs. This is particularly important given the scarcity of resources and time constraints in these countries. Large-scale, well-designed studies should be conducted to explore the development and implementation of contextually relevant validated screening tools such as the SADS.

To ensure that the identified barriers in resource-constrained countries are accounted for, it is essential to conduct context-specific research. Moreover, studies exploring the implementation of supportive strategies and education to improve nurses’ skills in dysphagia care should be conducted. Such studies should focus on improving nurses’ skills and not exploring just their perceptions or experiences of working in a team. Overall, these areas require further investigation to improve the quality of care for patients with stroke-related OPD.

## Conclusion

The scoping review has identified a clear need for further training for nursing practitioners to improve knowledge and skill acquisition in the management of stroke-related OPD, as well as the benefits of collaboration between nurses and SLTs in LMICs. To address these needs, future research endeavours should focus on offering creative solutions to improve education and collaboration in dysphagia care in resource-constrained settings. Rather than solely evaluating nursing practices and perceptions, future research should target the identified needs of nurses to improve dysphagia care in LMICs.

## Appendix 1

**TABLE 1-A1:** Number of results for each search phrase obtained from the various databases.

Search terms	Number of responses (selected fields)						Total
	PubMed (title/abstract)	EBSCOhost CINAHL (all fields)	Scopus (title/abstract/*keywords*)	Web of Science Core Collection (all fields)	EBSCOhost Health source: Nursing and academic edition (all fields)	Cochrane Libraries (title/abstract/*keywords*)	
(knowledge) AND (nurse) AND (stroke) AND (dysphagia)	18	1603	17	22	5	2	1667
(knowledge OR education OR understanding OR awareness OR training) AND (nurse) AND (stroke) AND (dysphagia)	3 691 195	41	62	5 091 259	9	18	8 782 584
(knowledge OR education OR understanding OR awareness OR training) AND (nurse) AND (stroke OR cerebrovascular accident OR CVA) AND (dysphagia OR swallowing disorders OR deglutition disorders)	4 060 286	48	26	5 104 135	9	25	9 164 529
(practices) AND (nurse) AND (stroke) AND (dysphagia)	50	46	59	62	20	1	238
(knowledge) AND (nurse) AND (stroke) AND (dysphagia) AND (low- and middle-income countries)	1	1	1	1	0	0	4
(collaboration) AND (nurse) AND (stroke) AND (dysphagia)	10	3	6	6	3	1	29

**Total number of results**	**7 751 560**	**1742**	**171**	**10 195 485**	**46**	**47**	**17 949 051**
